# ERBB3: A potential serum biomarker for early detection and therapeutic target for devil facial tumour 1 (DFT1)

**DOI:** 10.1371/journal.pone.0177919

**Published:** 2017-06-07

**Authors:** Dane A. Hayes, Dale A. Kunde, Robyn L. Taylor, Stephen B. Pyecroft, Sukhwinder Singh Sohal, Elizabeth T. Snow

**Affiliations:** 1 Department of Primary Industries, Parks Water and Environment, Animal Health Laboratory, Launceston, Tasmania, Australia; 2 Save the Tasmanian Devil Program, University of Tasmania, Hobart, Tasmania, Australia; 3 School of Health Sciences, Faculty of Health, University of Tasmania, Launceston, Tasmania, Australia; 4 Department of Primary Industries, Parks Water and Environment, Resource Management and Conservation, Hobart, Tasmania, Australia; 5 School of Animal & Veterinary Sciences, Faculty of Science, University of Adelaide, Roseworthy Campus, Roseworthy, South Australia; University of South Alabama Mitchell Cancer Institute, UNITED STATES

## Abstract

Devil Facial Tumour 1 (DFT1) is one of two transmissible neoplasms of Tasmanian devils (*Sarcophilus harrisii*) predominantly affecting their facial regions. DFT1’s cellular origin is that of Schwann cell lineage where lesions are evident macroscopically late in the disease. Conversely, the pre-clinical timeframe from cellular transmission to appearance of DFT1 remains uncertain demonstrating the importance of an effective pre-clinical biomarker. We show that ERBB3, a marker expressed normally by the developing neural crest and Schwann cells, is immunohistohemically expressed by DFT1, therefore the potential of ERBB3 as a biomarker was explored. Under the hypothesis that serum ERBB3 levels may increase as DFT1 invades local and distant tissues our pilot study determined serum ERBB3 levels in normal Tasmanian devils and Tasmanian devils with DFT1. Compared to the baseline serum ERBB3 levels in unaffected Tasmanian devils, Tasmanian devils with DFT1 showed significant elevation of serum ERBB3 levels. Interestingly Tasmanian devils with cutaneous lymphoma (CL) also showed elevation of serum ERBB3 levels when compared to the baseline serum levels of Tasmanian devils without DFT1. Thus, elevated serum ERBB3 levels in otherwise healthy looking devils could predict possible DFT1 or CL in captive or wild devil populations and would have implications on the management, welfare and survival of Tasmanian devils. ERBB3 is also a therapeutic target and therefore the potential exists to consider modes of administration that may eradicate DFT1 from the wild.

## Introduction

The Tasmanian devil (*Sarcophilus harrisii*) belongs to the Dasyuridae family, it is a carnivorous marsupial that is extinct on mainland Australia and now found only on the island of Tasmania. Superficial dermal cutaneous lesions of wild Tasmanian devils can be found commonly in the form of skin sores [1] and neoplasia [2]. Spontaneous neoplasms in captive Tasmanian devils including squamous cell carcinoma of the lip and gingiva, dermal lymphosarcoma [3], trichoepithelioma, papilloma and keratoacanthoma [4] and a single devil with multiple unrelated tumours involving internal organs in combination with skin [5] have been recorded, suggestive of potential metastasis. Similar observations were made while reviewing Dasyurid archival material at the Australian Registry of Wildlife Health [6] and recently, two captive female devils with pruritus and dermatitis were diagnosed with cutaneous T-cell lymphoma [7]. None of the recorded neoplastic superficial lesions found in captive or wild Tasmanian devils appeared to mimic the firm, flattened centrally ulcerated soft tissue lesions of DFT1 affected Tasmanian devils [8].

Although the first evidence of DFT1 in wild populations occurred in 1996 when several Tasmanian devils were photographed by Christo Baars in the north east of the state with facial lesions. However, a tissue diagnosis was not obtained until 2001 [9]. Review of Tasmanian devil archival slides submitted to the Animal Health Laboratory, DPIPWE, revealed a single case in 1997 that was consistent with DFT1 [8, 10]. An emerging disease was finally recognised in 2003 [10] and subsequent investigations revealed the tumour to be a transmissible allograft being transferred from devil to devil via biting [11] with tumours tending to be located on the face, lips and oral mucosa [8].The timeframe of the pre-clinical stage of DFTD1 remains largely undetermined with observations ranging from 2–13 months [9, 12–15] but as little as 1 month has been recorded (Author unpublished observation, laboratory records, DPIPWE). Immunohistochemical examination of DFT1 suggested a possible undifferentiated neuroendocrine tumour [16, 17] although subsequent molecular testing lead to the conclusion that DFT1 is of Schwann cell origin [18]. Down-regulation mechanisms causing absence of major histocompatibility complex (MHC) class 1 cell surface antigens is a major contributing factor allowing the DFT1 allograft to evade the host devil’s immune system without rejection [19–21]. Further cytogenetic and molecular techniques have identified four karyotypic strains that are differentiated by a small number of identifiable rearrangements [22, 23]. As a consequence of this cancer, wild populations of the Tasmanian devil have been significantly reduced in Tasmania where the possibility of extinction either locally within 10–15 years [24, 25] or completely within 25–35 years [25] has been predicted. The impedance of this 2007 dire prediction includes the adaption of wild Tasmanian devils to their life history change by precocial sexual maturity [26] and through a strong collaborative scientific research and conservation management framework devised by the Save the Tasmanian Devil Program (STDP) [27]. A second transmissible tumour in Tasmanian devils, devil facial tumour 2 (DFT2), distinct from DFT1 has recently been reported [28] suggesting that the species may well be prone to transmissible cancers, increasing the urgency of biomarker identification and therapeutic intervention.

ERBB3 is expressed in early embryonal development and plays an integral role in the development of the neural crest and Schwann cells [29] regulating pathways that execute diverse cellular functions including development, cell cycle, migration, survival, proliferation and differentiation [30–34]. ERBB3 is a member of the Epidermal Growth Factor (EGF) family representing a complex group of type 1 transmembrane receptor tyrosine kinase (RTK) with differing ligands. The EGF family consists of four members and collectively the human epidermal growth factor receptor gene family members are designated *EGFR/ERBB1/HER1*, *ERBB2/HER2*, *ERBB3/HER3* and *ERBB4/HER4* [35]. The extracellular domain (ECD) of ERBB receptors has high structural homology although they bind selectively within a group of 11 peptide growth factor members that includes Neuregulin 1 and 2 (NRG1/NRG2) both ERBB3 ligands. [35–39]. Although the complex signalling network of ERBB receptors commonly activate the mitogen activated protein kinase (MAPK) pathway and the phosphatidylinositide 3-Kinase (PI3K) pathway [40–43], ERBB3 efficiently activates the PI3K pathway [44] due to the presence of multiple p85 binding sites in its tyrosine kinase domain.

Lateral signalling among ERBB’s is no more apparent than with receptors ERBB2 and ERBB3 that must heterodimerise with other ERBB members to signal [40] as ERBB3 has a ligand but impaired tyrosine kinase activity [45] and ERBB2 has no known ligand (orphan receptor) but a functional kinase region [46]. Although ERBB3 has long been considered impaired or termed a pseudo-kinase, it does have sufficient, although substantially reduced [47], kinase activity. How ERBB3 is able to activate other ERBB family members with its weak catalytic domain remained elusive until an allosteric mechanism termed an ‘asymmetric dimer’ enabling trans-autophosphorylation was discovered [48].

ERBB2 and ERBB3 overexpression [49–51], cooperation in neoplastic transformation [44, 52–54] and loss of ERBB3 preventing the progressive transformation of ERBB2-over expressing tumours [55] reinforces ERBB3’s pivotal role in ERBB signalling. Early studies revealed ERBB3 as a potential oncogene with overexpression due to possible increased transcription as no gene amplification was observed [56, 57] although recently oncogenic mutations have been reported [58] indicating either ERBB3 or its downstream components should represent a potential target for therapy [59].

ERBB3 is upregulated in a number of human cancers such breast, colon, gastric, ovarian and prostate [33, 60] but seldom reported in veterinary cancers [61–63] although it would appear the instrumental role that ERBB3 may play in some veterinary tumours is yet to be elucidated. DFT1’s immunohistochemical expression of ERBB3 led us to postulate that excess extracellular domain (ECD) may circulate in the host’s plasma and present itself as a possible candidate biomarker for DFT1. Literature reports five secreted alternative transcripts of ERBB3 present in serum or interstitial fluid [64, 65] which can be detected utilising ELISA methodology.

Our pilot study assessed serum ERBB3 for the for the first time in Tasmanian devils revealing that serum ERBB3 was substantially elevated in the serum of Tasmanian devils with DFT1 compared to those Tasmanian devils without DFT1. Interestingly, the inclusion of some Tasmanian devils with CL in our pilot study revealed that ERBB3 may also be a biomarker for this DFT1, although CL is clinically distinct from DFT1. We identify ERBB3 as a potential biomarker of DFT1 and highlight current literature supporting the therapeutic possibilities that can be directed towards ERBB3 overexpressing tumours that may be helpful in the elimination of DFT1 from the wild.

## Materials and methods

### Animal ethics statement

Serum and paraffin embedded tissue samples were collected by veterinary staff for the Save the Tasmanian Devil Program (STDP) http://www.tassiedevil.com.au/tasdevil.nsf encompassing health checks, field trapping trips, or autopsy due to animal welfare reasons. All samples were accessed from the Animal Health Laboratory archive and did not require ethics approval.

### Tasmanian devil ERBB3 pilot study

A pilot study of thirty-five Tasmanian devils differing in age, sex and geographic location were selected (Table 1) to compare serum ERBB3 levels in clinically healthy Tasmanian devils (CHD), devils with DFT1 and those with CL. The Fifteen CHD’S included both adults (n = 12) and clinically healthy juvenile Tasmanian devils (CHJD, n = 3) 10 months of age. Adults included free range captive (n = 5), captive (n = 3) and wild devils (n = 4). Clinically healthy adults either had no visible disease (ND, n = 8) or had localised skin non-DFT1 dermatopathy (CHDD, n = 4) consisting of two abscesses, a skin tag and localised dermatitis. Eight Tasmanian devils with clinical DFT1 and Twelve Tasmanian devils with CL. Tasmanian devils with CL were included in the study as a severe skin condition recognised clinically but very distinct from DFT1. All dermatopathies, DFT1 and CL were confirmed histologically by the Animal Health Laboratory.

**Table 1:** Tasmanian devil pilot study individuals.

Devil	Microchip Identification	Laboratory accession	Age (years)	Sex (M/F)	Geographic location	Clinical status
1	982000190997443	13/3712	1	F	Freycinet ^a^	CHD
2	982000123211124	13/3683	3	F	Freycinet ^a^	CHD
3	982009104963600	13/3680	4	M	Freycinet ^a^	CHD
4	982009104860765	13/3713	4	M	Freycinet ^a^	CHD
5	982000123130282	13/3716	2	M	Freycinet ^a^	CHD
6	982009105111670	09/4200	3	F	West Pencil Pine ^b^	CHD
7	982009105849999	09/3957	2	M	Tullah ^b^	CHD
8	985154000001063	09/1051	1	M	Cressy ^c^	CHD
9	982009104269684	08/1805	2	M	Narawntapu ^b^	CHDD
10	982009106039877	10/0156	2	M	Dunalley ^b^	CHDD
11	982009104236464	08/0798	1	F	Taroona ^c^	CHDD
12	982009104357109	09/2009	4	F	Fern Tree ^c^	CHDD
13	985154000001151	09/0451	<1	M	Mt Pleasant ^d^	CHJD
14	985154000001142	09/0449	<1	F	Mt Pleasant ^d^	CHJD
15	985154000001130	09/0448	<1	M	Mt Pleasant ^d^	CHJD
16	982009104841875	12/2065	6	F	West Pencil Pine ^b^	DFT1
17	982009106034139	11/0767	2	F	Dunalley ^b^	DFT1
18	982009104719592	12/0820	4	F	West Pencil Pine ^b^	DFT1
19	982000000122095	12/2095	2	F	Upper Natone ^b^	DFT1
20	982000123128645	11/3917	2	M	Hamilton ^b^	DFT1
21	982000123216973	11/3918	1	F	Hamilton ^b^	DFT1
22	982000123209814	11/4493	2	M	Waratah ^b^	DFT1
23	000000000130406	13/0406	2	F	Mangalore ^b^	DFT1
24	NC	11/0650	7	F	Mole Creek ^c^	CL
25	985120016024404	11/4290	8	F	Mt Pleasant ^c^	CL
26	982009106314654	10/4001	8	M	Taranna ^c^	CL
27	982009106585887	10/3765	5	F	Calder ^b^	CL
28	982009104789818	14/0034	6	F	Cressy ^c^	CL
29	NC	08/4048	4	F	Circular Head ^b^	CL
30	982009100786171	09/0402	6	F	Mt Pleasant ^c^	CL
31	982009101694833	10/1013	6	F	Richmond ^c^	CL
32	982009104910854	13/0518	6	F	Cressy ^c^	CL
33	NC	09/3035	5	F	South Riana ^b^	CL
34	NC	11/1615	6	F	Mole Creek ^c^	CL
35	982009104873582	13/3714	4	F	Freycinet^a^	CL*

NC not microchipped, CHD clinically healthy devil, CHDD clinically healthy devil with dermatopathy, CHJD clinically healthy juvenile devil, DFT1 devil facial tumour 1, CL cutaneous lymphoma

^a^ Free range enclosure

^b^ Wild devil

^c^ Captive devil

^d^ captive juvenile

* no tissue diagnosis.

### Tasmanian devil serum sample and collection

Blood samples from Tasmanian devils (Table 1) were collected by wildlife veterinarians through jugular venepuncture, whilst the animals were restrained by a trained field officer. Ten millilitres of blood was collected in sterile serum separation tubes, stored on ice for transport to the laboratories, centrifuged and serum removed for archival storage at -20^°^C. Serum samples were retrieved from the frozen archive and thawed at room temperature immediately before analysis.

### Histology

Tasmanian Devil tissues were fixed in 10% Neutral Buffered Formaldehyde (Confix, ACFC, Australian Biostain, Traralgon, Victoria, Australia) for 24 hours and selected tissues were cassetted and processed overnight using a standard 15 hour overnight procedure in the TP1050 tissue processor (Leica Microsystems, Wetzlar, Germany). Tissues were orientated on the EG1160 (Leica), embedded in paraffin wax (Surgipath Paraplast, 39601006, Leica) and sectioned at 3 microns using Leica RM2245 microtome and adhered to microscope slides (Menzel Glaser, Braunschweig, Germany) for 20 minutes at 60^°^C. Sections were deparaffinised, rehydrated and stained using Jung autostainer XL (Leica) for Haematoxylin (Harris’ Haematoxylin, AHHNA, Australian Biostain) and Eosin, dehydrated cleared and mounted in CV Mount (Leica, 046430011).

### Immunohistochemistry

Archival Tasmanian devil tissues and tumours were sectioned at 3 microns, floated onto Superfrost plus slides (Menzel Glaser) and subjected to standard deparaffinisation and rehydration techniques using an automated stainer (Leica). Antigen retrieval in tissue sections was conducted in citrate buffer at pH 6.0 (Reveal Decloaker, Biocare Medical, California, USA) at 120^°^C for 8 minutes using a Pascal pressure chamber (Dako, Glostrup, Denmark) then cooled to 20^°^C. Endogenous peroxidase activity was quenched using 3% hydrogen peroxide (Ajax Finechem, Sydney, Australia, 260) in methanol (Ajax, 723) for 30 minutes. Detection of primary antibodies was achieved using Mach1 Universal HRP-Polymer detection kit (Biocare Medical, California, USA, M1U539GL10). Protein block (Background Sniper BS966L10) was applied for 20 minutes prior to application of primary antibodies. Monoclonal rabbit anti-human ERBB3 (Abcam, clone SP71, ab93739, internal region) was diluted 1:50 with antibody diluent (Dako, S0809) and applied to both devil tumour and normal devil control tissues at room temperature for 30 minutes. Negative control was omission of primary antibody with buffer substitution. Universal HRP-polymer was applied for 30 minutes (MRH538L10) followed by 1 drop of Betazoid DAB Chromogen 3,3 Diaminobenzidine (BDB900G) in 1ml of substrate buffer (DB900) applied for 4 minutes. Tris buffered saline (Biocare Medical, TWB945) was used to rinse between all steps. Slides were rinsed, stained with Carazzi’s Haematoxylin for 5 minutes, washed for 3 minute in tap water, dehydrated, cleared and mounted in CV mount. Sections were viewed under light microscopy using Olympus BX41 (Olympus corporation, Tokyo, Japan) and selected areas were photographed using an Olympus digital camera (DP20).

### ERBB3 ELISA assay

Serum ERBB3 levels were measured using the RayBio anti-human ERBB3 ELISA Kit (ELH-ERBB3, RayBiotech Inc, GA, USA) according to manufacturer’s instructions. Briefly, serum samples were diluted 1/5 in Assay Diluent A and 100 uL of standard or diluted sample were added in duplicate to wells of a 96 well assay plate and incubated for 24 hrs at 4^°^C. The supernatant was removed and wells were washed 4 times with 300 uL of 1X wash solution using an Immunowash 1575 (BioRad Laboratories, CA, USA). One hundred microliters of prepared biotinylated anti-ERBB3 was added to each well and the assay plate incubated for 1 hour at room temperature. The assay plate was washed as described after which 100 uL of prepared HRP-streptavadin conjugate was added to each well and the assay plate incubated for 45 minutes at RT. The assay plate was again washed as described and 100 uL of TMP substrate was added and the plate incubated for 30 minutes at room temperature in the dark, after which 50 uL of stop reagent was added to each well. The absorbance of each well was measured at 450 nm using a Tecan Infinite M200 microplate reader (Tecan, Salzburg, AUT).

### Data analysis

The ELISA standard curve was plotted using Prism v5 (GraphPad, CA, USA) and results for each serum interpolated and corrected for dilution. The significance of differences in serum ERBB3 between groups was determined using a Kruskal-Wallis test with Dunn’s Multiple Comparison utilizing Prism v5 (GraphPad, CA, USA).

## Results

### Histology and Immunohistochemstry

**Fig 1:**
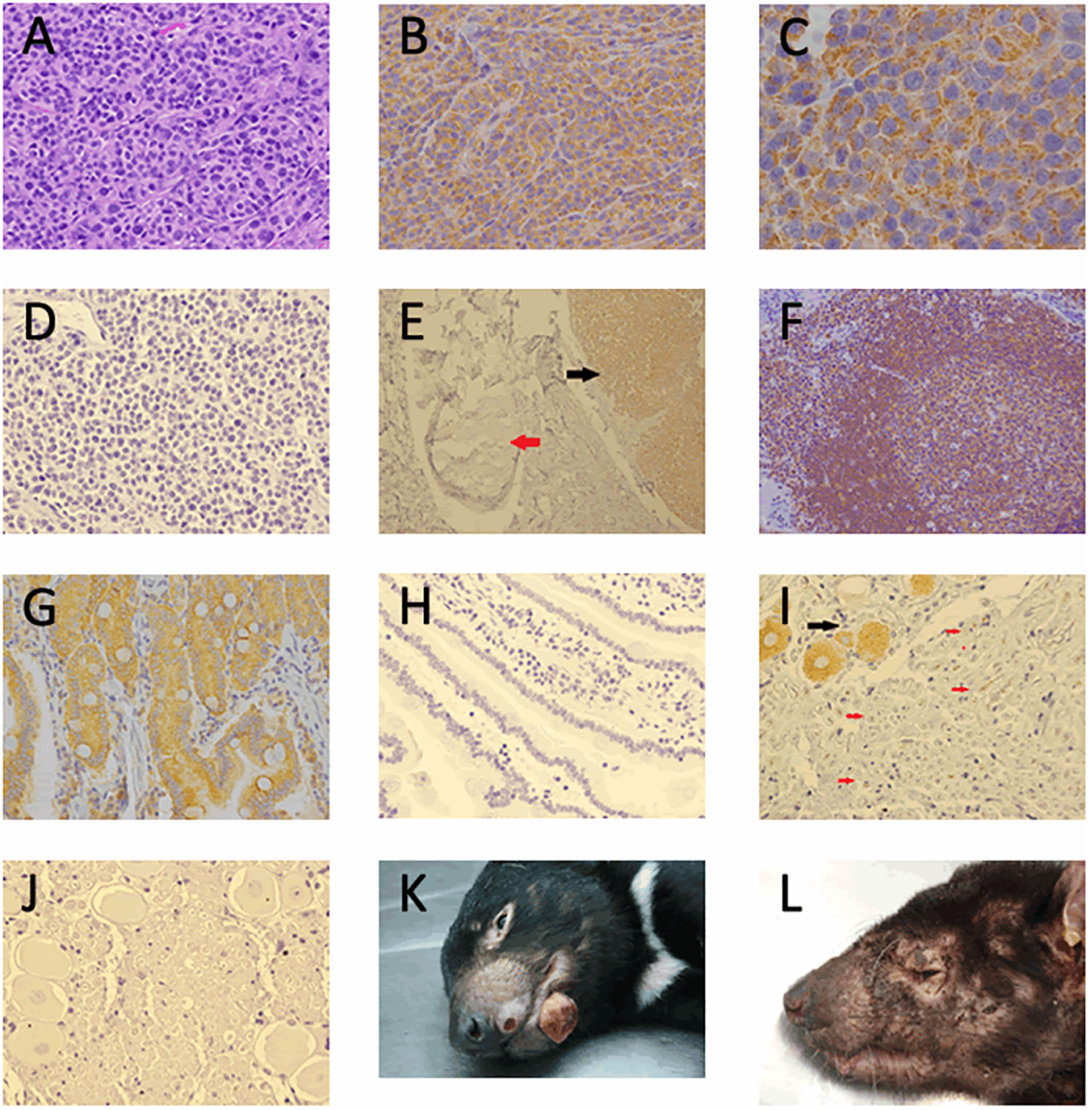
DFT1 staining and skin manifestation. **(A)** Haematoxylin and Eosin stained DFT1 x40, (**B)** ERBB3 Immunohistochemical expression in DFT1 strain 3 x40, (**C)** ERBB3 immunohistochemical expression in DFT1 strain 3 x100, (**D)** DFT1 negative control, (**E)** Tasmanian devil skin and subcutis section with peripheral nerve (red arrow) and DFT1 (black arrow) x10, **(F)** Tasmanian devil lymph node ERBB3 expression lymphoid follicle x20, (**G)** Tasmanian devil bowel ERBB3 positive control x40, (**H)** ERBB3 IHC negative control bowel, (**I)** trigeminal nerve shows ERBB3 positive nerve body (black arrow) and occasional adaxonal ERBB3 positivity (red arrows) x40, (**J)** ERBB3 IHC negative control trigeminal nerve, (**K)** Tasmanian Devil gross appearance of DFT1. Photo credit: DPIPWE archive, (**L)** Tasmanian devil gross appearance cutaneous lymphoma. Photo credit DPIPWE archive.

DFT1 histology (Fig 1A) and Haematoxylin and Eosin demonstrates small round cells with indistinct cell membranes arranged in cords and packets. ERBB3 IHC on average revealed moderate to strong expression in 75% of cells in both primary and secondary DFT1 tumours in cytogenetically determined strains 1 to 5 of DFT1. Typical granular cytoplasmic expression (Fig 1B) demonstrated by DFT1 strain 3 cells with small and large aggregates noted. Higher magnification (Fig 1C) shows accumulation in and around vacuolar structures within the cytoplasm. In sections of devil skin and subcutous (Fig 1E), peripheral nerve was seldom positive for ERBB3 (red arrow) in keeping with downregulation of ERBB3 in the adult in contrast to DFT1 ERBB3 expression (black arrow). ERBB3 expression was noted in Tasmanian devil lymphoid follicle (Fig 1F) where cytoplasmic expression of ERBB3 is present in both T (germinal centre) and B (mantle) cells. Devils with CL were not included in the ERBB3 immunohistochemical staining. Trigeminal nerve section (Fig 1I) showed ERBB3 expression in nerve bodies (black arrow) and occasional ERBB3 expression in the adaxonal area (red arrows) but generally small myelinated nerves were negative. Positive control included devil bowel (Fig 1G) which exhibited a similar expression pattern to human ERBB3 and negative controls DFT1 (Fig 1D), bowel (Fig 1H) and Trigeminal nerve (Fig 1J). The monoclonal rabbit anti-human ERBB3 clone SP71 is a synthetic peptide corresponding to an internal sequence of Human ERBB3. Although the exact sequence is a proprietary secret ERBB3 sequence alignment between Human and Tasmanian devil in this region has high homology (S1 Fig. ERBB3 Orthologue protein alignment).

### Serum ERBB3 in Tasmanian devils

**Fig 2:**
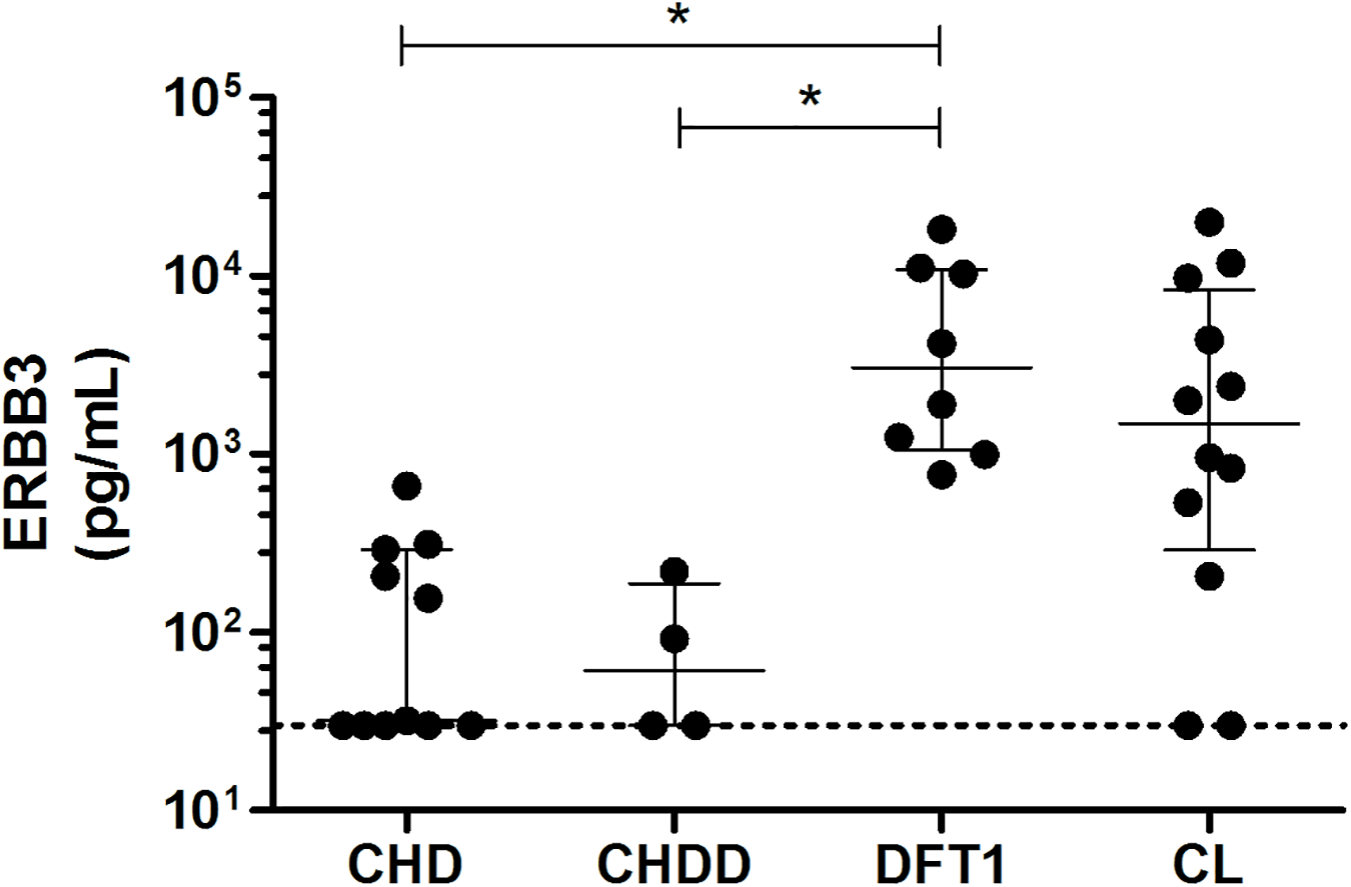
Serum ERBB3 levels in Tasmanian devils. Serum ERBB3 levels were measured by ELISA in clinically healthy Tasmanian devils CHD (n = 11), clinically healthy Tasmanian devils with dermatopathy CHDD (n = 4), clinically diagnosed DFT1 (n = 8) and those with cutaneous lymphoma CL (n = 12). Horizontal dashed line indicates the limit of detection of the ELISA assay at 30 pg/mL. Results of individual devils are shown with the median and interquartile range identified by the whiskers. Significance testing using a Kruskal-Wallis test with Dunn’s Multiple Comparison Testing shown with * representing p < 0.05.

Serum ERBB3 levels are shown in Table 2 and graphically in Fig 2. Serum ERBB3 in the Fifteen Tasmanian devils without neoplasia (devils 1–15 includes CHD,CHDD and CHJD) ranged from <30–663 pg/ml with a median of 32 pg/mL (30–220; interquartile range). Serum ERBB3 levels in the eight Tasmanian devils (devils 16–23) with clinical DFT1 ranged from 766–18,254 pg/ml with median of 3051 pg/mL (1060–10879; interquartile range. In the twelve Tasmanian devils with cutaneous lymphoma (devils 24–35) serum ERBB3 levels ranged from <30–20,021 pg/ml with a median of 1485 pg/mL (289–7901; interquartile range).

**Table 2:** Tasmanian devil serum ERBB3 and clinical history.

Devil	Serum Erbb3 (pg/ml)	Weight (Kg)	Serum transit (days)	clinical history	BCS (0–5)	DFT1 strain	DFT1 1^o^ N^o^ (range cm)	Mets N^o^
1	155	N/A	1	CHD, NAD				
2	663	N/A	1	CHD, Localised alopecia				
3	207	OW	1	CHD, Multiple punctures				
4	313	N/A	1	CHD, Multiple punctures				
5	291	N/A	1	CHD, Multiple minor wounds				
6	<30	N/A	1	CHD, Few wounds, lactating				
7	<30	N/A	2	CHD, N/A				
8	<30	6	1	CHD, Great condition				
9	220	10.5	1	CHDD, Abscess/scab on face				
10	92	N/A	2	CHDD, Abscess left neck.				
11	<30	4.7	1	CHDD, Skin tag on left ear				
12	<30	N/A	1	CHDD, Dermatitis upper flank				
13	<30	4.2	1	CHJD, Health check				
14	32	3.4	1	CHJD, Health check				
15	<30	4.6	1	CHJD, Health check.				
16	18,254	N/A	1	DFT1, weak	2	2	2 (1.0–2.5)	3
17	999	6.1	3	DFT1, Reared 4 young	2	3	4 (1.0–1.5)	5
18	11,090	4.8	1	DFT1, Poor body condition	1–2	1	4 (2.0–3.0)	1
19	1903	3.7	1	DFT1, Emaciated disorientated	0	1	2 (1.6–5.2)	10
20	10,247	10	3	DFT1, Multiple lesions	3	2	4 (1.0–2.0)	2
21	1241	5	3	DFT1, Poor body condition	2.5	2	3 (1.0–1.5)	1
22	4198	9.3	4	DFT1, Advanced DFT1	2	4	7 (1.0–2.5)	0
23	766	N/A	1	DFT1, Emaciated	2	1	7 (1.0–4.7)	1
24	4383	6.7	1	CL, Generalised alopecia	N/A			
25	<30	8.2	1	CL, cutaneous plaques chest	N/A			
26	<30	8.0	1	CL, percutaneous plaque	N/A			
27	2008	5.9	1	CL, Skin lesions	N/A			
28	837	5.9	1	CL, Alopecia	Poor			
29	9703	5.3	1	CL, Generalised alopecia	Poor			
30	2403	8.2	1	CL, Alopecia ventrally	N/A			
31	536	7.4	1	CL, Alopecia left neck, pouch	N/A			
32	962	6.7	1	CL, Alopecia ventrally	N/A			
33	11,837	5.4	1	CL, Widespread alopecia	1–2			
34	207	5.7	1	CL, Multifocal dermatitis, cutaneous lump (acanthoma)	Poor			
35	20,021	N/A	1	CL, Multifocal alopecia	N/A			

N/A not available, NAD no abnormality detected, OW over weight, BCS—body condition score, DFT1 strain–cytogenetically determined strain, DFT1 1^o^ No–number and size of primary tumours recorded, Mets No—number of metastasis recorded, CHD clinically healthy devil, CHDD clinically healthy devil with dermatopathy, CHJD clinically healthy juvenile devil, DFT1 devil facial tumour 1, CL cutaneous lymphoma

## Discussion

### ERBB3 in devils without DFT1

Fifteen Tasmanian devils without neoplasia (twelve adults either wild caught, free range or captive enclosures and three captive juveniles encompassing CHD, CHDD and CHJD) were studied with an average serum ERBB3 of 32 pg/ml. Collectively, CHD Tasmanian devils serum ERBB3 levels ranged from <30–663 pg/ml which could be considered representative of the reference range for Tasmanian devils. Wild caught devils 6 and 7 were unremarkable and had serum ERBB3 levels <30 pg/ml however devil 9 (220 pg/ml) and devil 10 (92 pg/ml) both recorded skin abscesses. The ERBB3 levels in the CHDD group (devils 9, 10, 11 and 12) ranged from <30–220 pg/ml all had a small isolated dermatopathy such as abscess (devil 9), pyogranuloma (devil 10), skin tag with associated inflammation (devil 11) and small focus of dermatitis (devil 12) all recorded a low serum ERBB3 levels of <92 pg/ml. The CHJD (devils 13, 14 and 15) approximately 10 months old had an unremarkable clinical history that indicated serum was collected for a health check only, reflected in the low serum ERBB3 level of <30 pg/ml.

Further assessment of data and clinical history (Table 2) revealed that four out of five Tasmanian devils from the Freycinet free range enclosure (devils 1–5) had higher serum ERBB3 ranging from 155–663 pg/ml compared to most other clinically healthy devils having serum ERBB3 levels <30 pg/ml. The Freycinet free range enclosure (FRE) consists of a 22 Hectare natural reserve that creates living conditions that are more similar to the wild than traditional captive conditions. The structure is fenced completely enclosing an insurance population of healthy devils with density caped to approximately one devil per hectare. This type of enclosure allows devils the opportunity to compete at feeding and breeding times and bite wounds are therefore common (David Schaap, personal communication). In contrast, captive devils are housed in small enclosures that measure approximately 100 m^2^ containing capped at one devil per 100 m^2^.

We noted that skin injuries were commonly recorded although no abnormality was noted for devil 1, alopecia bilaterally around the hind limbs and flank was present on one mother due to her 3 pouch young (devil 2) and multiple puncture wounds were present on the remainder (devils 3, 4 and 5). Given that these devils were otherwise clinically healthy it would suggest that skin wounds caused by biting may contribute to some elevation in the serum ERBB3 of Tasmanian devils. There is also the possibility that simply being a Tasmanian devil living in a free range enclosure as opposed to wild populations may in itself be contributory to elevation in serum ERBB3 due to more frequent devil-devil engagement. Our results indicate that Tasmanian devils without injuries or an isolated skin lesion have serum ERBB3 levels <30 pg/ml whereas Tasmanian devils with multiple injuries or large abscesses have serum ERBB3 levels ranging from 92–663 pg/ml. Together, these results suggest that cancer-free Tasmanian devils have a serum ERBB3 range of <30–663 pg/ml.

### ERBB3 in devils with DFT1

All devils with DFT1 were wild caught and all subjected to field autopsy with most serum samples reaching the laboratory within one to three days. We assessed the available clinical history (Table 2) including animal weight, body condition score (BCS 1–5) where 1 = emaciated, 2 = moderately thin, 3 = average, 4 = good and 5 = obese (Sarah Peck, personal communication), number of primary and metastatic DFT1’s and cytogenetic strain ensuring the consideration of any factors that may contribute to the ERBB3 range in DFT1 affected Tasmanian devils. No correlation was established between levels of ERBB3 and extent of DFT1 when comparing the number and size of primary DFT1 lesions and any metastatic disease (see Table 2). For example, the devil with the highest serum ERBB3 of 18,254 pg/ml (devil 16), had 2 primary lesions with 3 metastases whereas the lowest serum ERBB3 of 766 pg/ml (devil 23) had seven primary DFT1 lesions and one metastasis. No correlation was established between serum ERBB3 levels and the BCS as most were low (BCS 1–2) with only one devil (devil 20) having a BCS of three out of five, indicating average body condition. Cytogenetic strain did not appear to correlate to serum ERBB3 levels and reflects the immunohistochemical findings that ERBB3 expression was present in all cytogenetic strains of DFT1. Our results indicate that Tasmanian devils with DFT1 have elevated serum ERBB3 levels compared to clinically healthy Tasmanian devils ranging from 766–18,254 pg/ml and that the extent of DFT1 does not readily correlate directly with the serum ERBB3 levels. Further investigations beyond the pilot study encompassing a larger study group of Tasmanian devils with advanced DFT1 and metastases would be necessary to establish any relationship with serum ERBB3 and the extent of DFT1.

### ERBB3 in devils with cutaneous lymphoma

We included Tasmanian devils with cutaneous lymphoma (CL) in the study for two reasons. Firstly, they were non-DFT1 devils with a severe skin condition that can affect the facial regions and secondly, the disease presentation of alopecia, excoriation and thickened plaques is distinct from DFT1 (Fig 1E and 1F). Our results revealed that some Tasmanian devils with CL had elevated serum ERBB3 levels, a result that was most unexpected. Although ERBB3 immunohistochemistry on Tasmanian devils with CL was beyond the scope of this research, ERBB3 Immunohistochemical staining of Tasmanian devil lymph node (Fig 1D) did reveal ERBB3 expression in the lymphoid follicle where cytoplasmic expression of ERBB3 is present in both T (germinal centre) and B (mantle) cells. CL devils were in the older age bracket ranging from 4–8 years where the maximum age of a wild devil would be considered 5–6 years (Sarah Peck, personal communication). Bodyweights ranging from 5.4–8.2 Kg compared to the mean weight of 6.6Kg for female and 8.3Kg for male [66] shows possible female underweight wild devils and overweight captive devils. Age or weight did not appear to correlate to the broad range of serum ERBB3 of 30–20,021 pg/ml. Interestingly, 11 of the 12 devils with CL were female. We noted that devils with widespread alopecia (devils 24, 29, 33 and 35), did exhibit increased serum ERBB3 levels ranging from 4383–20,021 pg/ml, suggesting that the severity of CL manifesting clinically as widespread alopecia may contribute to increased serum ERBB3 levels. Together, the elevated serum ERBB3 results in devils with CL is unlikely to cause confusion with DFT1 as CL tends to affects devils in the older age group and the clinical signs of CL are also distinct from DFT1 in established disease. Additionally, if elevated serum ERBB3 levels in Tasmanian devils indicative of CL could be established (pre-clinical) this would improve the healthy captive breeding populations of Tasmanian devils to ensure survival of the species by excluding these devils from this program.

### Potential source of serum ERBB3

The capture and detection of antibody in our ELISA assay is selective for the extracellular domain (ECD) of transmembrane ERBB3 in serum or plasma, thus ERBB3’s ECD is cleaved and shed from the plasma membrane would be a natural assumption. In contrast the ERBB3 receptor is internalised, although very slowly, for negative regulation and inactivation [67–71] utilising pathways such as caveolin or micropinocytosis and clathrin-and caveolin independent pathways [72, 73]. ERBB3 has also been shown to be endocytosed independent of phosphorylation and without ligand in clathrin-dependent manner [74]. ERBB3 is degraded by proteasomes catalysed by two E3 ubiquitin ligases; NRDP1 (Neuregulin Receptor Degradation Protein -1) [75], now known as RNF41 (Ring Finger 41, E3 Ubiquitin Protein Ligase) [76–78], and NEDD4 (Neural Cell Precursor Expressed, Developmentally Down-regulated 4, E3 Ubiquitin Protein Ligase) [79] that regulate steady-state ERBB3 levels influencing NRG1 signalling.

Defective internalisation, recycling and degradation of cell surface proteins and ligands is an emerging feature of cancer [80]. It is therefore conceivable that DFT1 is subjected to the same dysregulation and inefficient degradation and recycling resulting in over expression of ERBB3 receptor at the plasma membrane and subsequent detectable levels of serum ERBB3. While dysregulated endocytosis, deregulation and recycling may theoretically account for excess ERBB3 ECD detectable in serum, secreted isoforms of ERBB3 must also be considered as an alternative explanation for the presence of excess ERBB3.

As well as functional transmembrane forms, secreted soluble forms of Epidermal Growth Factor Receptors have been well documented for ERBB1 [81–84], ERBB2 [85–88] and ERBB4 [89–91]. Alternative transcripts for ERBB3 resulting in naturally occurring soluble truncated isoforms including a 1.4 kb transcript of ERBB3 in gastric cancer cell lines [64] and an additional four novel transcripts (1.6, 1.7, 2.1, and 2.3kb) from ovarian cancer cell lines [65] encouraged researchers to identify these secreted isoforms of ERBB3 in Prostate [92–95], liver [96], breast [97, 98] and squamous cell carcinoma [99]. ERBB3 isoforms have also been expressed intracellularly in breast cancer cell lines [97] as well as in the nucleus of Schwann cells [100, 101], prostate [102–104] and breast [105, 106]. Secreted ERBB3 isoform p85 has been shown to inhibit the action of its ligand Neuregulin [98, 107], nuclear translocations act as co-transcriptional activators [108], possible post-translation modification and the tumour microenvironment are instructive to serum ERBB3 secretion from the cell [96] and functions yet to be determined.

The antigenic peptide used for this assay is located within the N-terminal domain of the full length ERBB3 protein. Full length ERBB3 translates into a 180 kDa protein whereas ERBB3 transcripts, created by intron read through and alternative polyadenylation signals result in serum ERBB3 isoforms translating into various proteins ranging in size from 22–75 kDa [109]. Secreted isoforms such as ERBB3-S (1.4kb, 140aa homologous to the N terminus and a 43aa unique carboxy terminal sequence) equates to approximately half of domain I, p50 (1.6kb, 351aa homologous to the N terminus and a 30aa unique carboxy terminal sequence) equates to domain I, II and some of domain III, p45 (1.7kb, 310aa homologous to the N terminus and a 2aa unique carboxy terminal sequence) equates to domain I, II and some of domain III, p85 (2.1kb, 519aa homologous to the N terminus and a 24aa unique carboxy terminal sequence) equates to domain I, II,III and some of domain IV, p75 (2.3kb, 474aa homologous to the N terminus and a 41aa unique carboxy terminal sequence) equates to domain I, II and III [64, 65, 109] ERBB3 isoforms have been detected by a number of methods such as immunoprecipitation [65, 97, 107], immunohistochemistry [92] and ELISA [94–96]. Isoforms that have been detected using ELISA assays include p45 sERBB3 utilising a capture antibody of sequence aa20-643 (detection antibody sequence was not recorded) [94, 95] and 40-50kDa secreted isoforms (possible p45/p50) utilising both capture and detection antibodies with a sequence aa20-643 [96]. The Raybio ELISA kit utilised in our research uses a capture and detection antibody of sequence aa20-643 (personal communication Raybio) which accounts for most of the extracellular domain of ERBB3 and therefore would be able to capture and detect both truncated isoforms as well as the transmembrane ERBB3.

The correlation of serum levels with disease severity and progression would be the foundation of a good biomarker [96] as well; the expected biomarker should be in excess when compared to clinically healthy individuals [81] or possess additional qualities such as theranostic and tertiary prevention [84]. The use of serum ERBB’s as an indicator of human cancer appears useful however, its prognostic and theranostic value remains controversial and continued investigations will be required [81–96, 99]. The development of a diagnostic test for preclinical DFT1 would assist in the field operations if individuals could be identified before they become infectious[110], therefore application of serum ERBB3 as a diagnostic biomarker of DFT1 has great potential. The simplicity of the ELISA Serum ERBB3 methodology is easily incorporated into routine batch testing or rapid turnaround of results for urgent cases if required. Our research suggests that serum ERBB3 can be used as a biomarker for DFT1 and CL irrespective of transmembrane or truncated forms being detected in the serum of affected animals and therefore the potential of serum ERBB3 as a biomarker of early DFT1 detection should be explored.

### Schwann cell neoplasms

ERBB3 is crucial to the sequential transition from precursor to immature and finally mature Schwann cells where ERBB3 is down-regulated as myelination proceeds [111]. The adult peripheral nervous system requires maintenance when injured and the NRG1/ERBB system is crucial to Schwann cell dedifferentiation, proliferation, and subsequent regeneration and remyelination where ERBB3 and NRG1 is upregulated and only switched off after axon regeneration illustrating the plasticity of the Schwann cell [112–114]. Peripheral nerve sheath tumours [neurofibroma, malignant peripheral nerve sheath tumours (MPNST)] and schwannoma arise from the Schwann cell lineage and can be genetically characterised as Neurofibromas (either dermal or plexiform) and MPNST’s [Neurofibromatosis 1 (NF1)], or Schwannomas [Neurofibromatosis 2 (NF2)], Schwannomatosis and Carney complex type 1. Although distinct characterisation of these complex diseases is possible, frequent overlapping features make diagnosis difficult and must also include other tumours with a Schwannian component such as Neuroblastic and Granular Cell Tumours [reviewed in [115–119]]. Veterinary Schwann cell neoplasms have been recorded [120–124] although ERBB3 expression in Schwann cell neoplasia has not previously been reported in veterinary literature. ERBB3 receptor has been expressed in human Schwann cell neoplasms including neurofibroma, MPNST, Schwannoma, neuroblastic [125, 126] and ganglioneuroma (GN) tumours [127]. Interestingly, the down regulation of MHC class 1 and 2 molecules in a MPNST cell line [128] contrasting normal expression [129, 130] may indeed be similar to the MHC class 1 downregulation of DFT1 [19–21] resulting in defective antigen processing and presentation of the malignant Schwann cell neoplasm.

### ERBB3 as a therapeutic target

Despite evidence for multiple resistance mechanisms for existing therapeutic targeting of ERBB1/2 [131–141] numerous researchers have over the last decade explored the potential of ERBB3 as a therapeutic target [reviewed in [33, 60, 142–150]] using monoclonal antibodies [57, 151–176], histone inhibitors [177], TKI [178], surrobodies [179], locked nucleic acid (LNA)-based ERBB3 antisense oligonucleotide (ASO) [180], peptide mimics and vaccine [181], anti-anginal drug [182] and disulphide disrupting agent [183].

However, managing wildlife disease is considerably more difficult than human disease because of limited data, the effect of the disease on the host and the transmission of disease within a dynamic population makes it difficult to model [184]. Previous efforts to eradicate DFT1 from wild populations by selective culling has proven unsuccessful because of the frequency-dependent transmission of DFT1 and the latency period [110, 184, 185]. TKI’s as a therapeutic approach may be limited due primarlily to the early observation that kinase region of ERBB3 had substantialy reduced activity, however cancer immunotherapy broadly categorised as passive (including monoclonal antibodies, Cytokines, adoptive cell transfer) or active (including therapeutic cancer vaccine, immune checkpoint inhibitors) remains optimistic [186–191]. Many of these successful human immunotherapeutics do hold similar promise in veterinary medicine [192–194] however, drug administration to wild Tasmanian devils is very different from the clinical setting of human and companion animals and therefore treatments such as adoptive cell transfer would be difficult to implement. The fact that DFT1 expresses tumour associated antigens (TAA’s) such as ERBB3 invites the application of monoclonal antibodies and therapeutic cancer vaccines as prospective treatments. The passive administration of monoclonal antibodies to ERBB3 primarily focused on blocking receptor epitopes are still experimental [57, 151–176] and any humanised anti-ERBB3 would certainly have to be become species specific (devil anti-ERBB3) to prevent adverse immunologic reactions [195]. Very few monoclonal antibodies have been developed in veterinary oncology although two caninised antibodies anti-ERBB1 [196] and anti-CD20 [197] show promise. Therapeutic cancer vaccination modalities applicable to wildlife include antigen delivery vaccines that utilise inactivated cancer cells (autologous or allogenic) or peptide vaccines that mimic antigen sequences. Results using an inactivated cancer cell vaccine trial (allogenic DFT1 cell line) are eagerly awaited (http://www.utas.edu.au/news/2015/10/16/19-world-first-trial-of-tasmanian-devil-vaccine-begins-in-the-wild/). Confidence that immunisation can be successful stems from research showing that Tasmanian devils have a competent immune system [21, 198–200] and can produce cytotoxic antibodies [14, 201]. An alternative antigen presentation modality to cancer cell vaccine is a peptide vaccine, where single or multiple amino acid sequences (long or short) representing a defined antigen is combined with adjuvant to elicit an immune response [202]. Development of just a single ERBB3 peptide vaccine can be found in the literature [181] however, peptide vaccines targeting ERBB1 [203, 204], ERBB2 [205–207] or both ERBB1/2 [208] including monoclonal antibody against tyrosine related protein 1 (TRP-1) and altered peptide sequence to gp100 for mouse melanoma [209] all show promise. Overcoming self-tolerance is a major hurdle, one such strategy is the use of Xenoantigens, that is the exact same antigen but from a different species that has considerable sequence homology, differing only by several amino acids which appear to the host as altered epitopes or as “altered self” and therefore tolerance can be broken causing a T-cell response against the endogenous self-antigen [210]. Veterinary xenogeneic vaccinations include a DNA plasmid vaccine encoding human Tyrosinase (TYR) [211] the only veterinary therapeutic tumour vaccine licensed by the United States department of Agriculture (USDA) for the use of oral and digital melanoma, now marketed as Oncept^TM^.

Recent investigations reveal that the tumour microenvironment of metastatic DFT1 expressed B7-H1 and DFT1 cell lines could upregulate B7-H1[212]. Immune-suppressive tumour microenvironment created by tumour cells that escape ‘immunoediting’ allowing tumour growth and proliferation [213] where certain checkpoint pathways will be used advantageously by tumour cells to confer immune resistance [214]. Hence, checkpoint blockades (monoclonal antibodies) targeting Programmed Cell Death 1 (PD1 or PDCD1) and its ligand PD-L1 (B7-H1) and Cytotoxic T Lymphocyte Antigen 4 (CTLA-4) are now attractive therapeutical targets [215]. Recent views consider cancer immunotherapy invaluable although a single treatment mode may be suitable for some cases, more combinatorial approach will be needed for others [216, 217].

Our research has highlighted ERBB3 as a potential therapeutic target however treatment of Tasmanian devils with DFT1 with therapeutic regimes such as chemotherapy and radiotherapy are impractical. However, a combinatorial approach using therapeutic cancer vaccines including inactivated allogenic DFT1 cancer vaccine, ERBB3 monoclonal antibody, ERBB3 Peptide or xenogeneic vaccine in combination with anti-immune checkpoint blockade therapy would be easier to implement in the field as well as providing a sustained immunological response against DFT1.

## Conclusion

ERBB3 had previously avoided scrutiny due to its kinase inactivity; however, ERBB3 has now been the subject of intense investigation over the past decade and is now recognised as a potent partner of the epidermal growth receptor family. ERBB3 upregulation during developmental, dedifferentiation and regenerative processes encapsulates the Schwann cell’s inherent plasticity and imparts certain characteristics of malignant transformation advantageous to transmission of DFT1. Our pilot study has shown for the first time that ERBB3 is consistently expressed immunohistochemically and that ERBB3 is also elevated in the serum of Tasmanian devils with advanced DFT1 and cutaneous lymphoma. Therefore, our research indicates that serum ERBB3 has the potential to be employed as a biomarker of DFT1 or CL in Tasmanian devils to assist conservationists in the management and welfare of Tasmanian devils and species survival. The simplicity of the ELISA Serum ERBB3 methodology is easily incorporated into routine laboratory batch testing and equally applied to include rapid turnaround of results for urgent cases. Extension of this research is necessary to include greater numbers of healthy Tasmanian devils both with and without visible injuries, devils with large and small DFT1 lesions as well as pre-clinical DFT1. This will firmly establish the normal reference range for serum ERBB3 from which potential pre-clinical DFT1 may be identified. In addition, ERBB3 is now recognised as a therapeutic target and therefore the potential exists to consider modes of administration in addition to existing whole cell vaccination such as ERBB3 monoclonal antibody, peptide or xenogeneic vaccines including checkpoint inhibitors. A combinatorial immunotherapeutic approach will enhance cytotoxic destruction, provide long term immunity from DFT1 and therefore eradicate this transmissible tumour from the wild.

## Supporting information

S1 Fig ERBB3 Orthologue protein alignment. (DOCX)
