# Supplementary material for: ERBB3: A potential serum biomarker for early detection and therapeutic target for devil facial tumour 1 (DFT1)
Source: PLoS One. 2017 Jun 7;12(6):e0177919. doi: 10.1371/journal.pone.0177919 (PMC5462353; doi:10.1371/journal.pone.0177919)
Supplement: S1 Fig — (DOCX) [file pone.0177919.s001.docx]

**S1 Fig. ERBB3 Orthologue protein alignment**

| **Species** | **Gene ID** | **Peptide ID** | **Peptide length** | **% identity (Protein)** | **% coverage** | **Genomic location** |
| --- | --- | --- | --- | --- | --- | --- |
| Human (*Homo sapiens*) | ENSG00000065361 | ENSP00000267101 | 1342 aa | 82 % | 98 % | 12:56079857-56103505 |
| Tasmanian devil (*Sarcophilus harrisii*) | [ENSSHAG00000005870](http://asia.ensembl.org/Sarcophilus_harrisii/Gene/Summary?db=core;g=ENSSHAG00000005870) | [ENSSHAP00000006754](http://asia.ensembl.org/Sarcophilus_harrisii/Transcript/ProteinSummary?peptide=ENSSHAP00000006754) | 1328 aa | 83 % | 99 % | [GL861720.1:186836-237967](http://asia.ensembl.org/Sarcophilus_harrisii/Location/View?db=core;g=ENSSHAG00000005870;r=GL861720.1:186836-237967) |

CLUSTAL W (1.81) multiple sequence alignment

ENSP00000267101_Hsap/1-1342 ---MRANDALQVLGLLFSLARGSEVGNSQAVCPGTLNGLSVTGDAENQYQTLYKLYERCE

ENSSHAP00000006754_Shar/1-1328 PEVMKGLL---VLGWLLHLVGGSELGSFQGGCP----GINV---AESQNETLHQLCGWCE

*:. *** *: *. ***:*. *. ** *:.* **.* :**::* **

ENSP00000267101_Hsap/1-1342 VVMGNLEIVLTGHNADLSFLQWIREVTGYVLVAMNEFSTLPLPNLRVVRGTQVYDGKFAI

ENSSHAP00000006754_Shar/1-1328 VVMGNLEIVLTGHNFDLSFLKCIREVTGYVLISMNEFSDLSLSNLRLIRGAQVYGRKFAI

************** *****: *********::***** *.*.***::**:***. ****

ENSP00000267101_Hsap/1-1342 FVMLNYNTNSSHALRQLRLTQLTEILSGGVYIEKNDKLCHMDTIDWRDIVRDRDAEIVVK

ENSSHAP00000006754_Shar/1-1328 FVSLNYNTNSSQALRQFCLNQLTEILAGGVYIERNDKLCHMDTSDVRDIVRDQQAEIVIK

** ********:****: *.******:******:********* * ******::****:*

ENSP00000267101_Hsap/1-1342 DNGRSCPPCHEVCKGRCWGPGSEDCQTLTKTICAPQCNGHCFGPNPNQCCHDECAGGCSG

ENSSHAP00000006754_Shar/1-1328 NNGKNCPPCHESCGGKCWGPGPGDCQTLTKTICAPQCNGHCFGPNPNQCCHDECAGGCGG

:**:.****** * *:*****. ***********************************.*

ENSP00000267101_Hsap/1-1342 PQDTDCFACRHFNDSGACVPRCPQPLVYNKLTFQLEPNPHTKYQYGGVCVASCPHNFVVD

ENSSHAP00000006754_Shar/1-1328 PRETDCFACRHFNDSGACVSLCPLPLVYNKLTFQLEPNPHTKYQYGGVCVASCPHNFVID

*::****************. ** **********************************:*

ENSP00000267101_Hsap/1-1342 QTSCVRACPPDKMEVDKNGLKMCEPCGGLCPKACEGTGSGSRFQTVDSSNIDGFVNCTKI

ENSSHAP00000006754_Shar/1-1328 HTSCVRACPGDKMEVEKNGLKMCEPCGGLCPKACEGTGSGSRFQTVDSSNIDGFVNCTKI

:******** *****:********************************************

ENSP00000267101_Hsap/1-1342 LGNLDFLITGLNGDPWHKIPALDPEKLNVFRTVREITGYLNIQSWPPHMHNFSVFSNLTT

ENSSHAP00000006754_Shar/1-1328 LGNLDFLITGLKGDPWHNIPALDPEKLSVFQTVREITGYLNIQSWPPHMHNFSVFSNLTT

***********:*****:*********.**:*****************************

ENSP00000267101_Hsap/1-1342 IGGRSLYNRGFSLLIMKNLNVTSLGFRSLKEISAGRIYISANRQLCYHHSLNWTKVLRGP

ENSSHAP00000006754_Shar/1-1328 IGGRSLYNLGFSLLIMKNLNVTSLGLRSLKEVSAGRIYISANKQLCYYHSLNWTRLLRGP

******** ****************:*****:**********:****:******::****

ENSP00000267101_Hsap/1-1342 TEERLDIKHNRPRRDCVAEGKVCDPLCSSGGCWGPGPGQCLSCRNYSRGGVCVTHCNFLN

ENSSHAP00000006754_Shar/1-1328 KEGRLDIKHNRLKKDCVAEGQVCDPLCSSGGCWGPGPGQCLSCRNYSREGVCVTQCNFLN

.* ******** ::******:*************************** *****:*****

ENSP00000267101_Hsap/1-1342 GEPREFAHEAECFSCHPECQPMEGTATCNGSGSDTCAQCAHFRDGPHCVSSCPHGVLGAK

ENSSHAP00000006754_Shar/1-1328 GEPREFANEDECFSCHPECQPVEGNVTCYGSGSDACAQCAHFRDGPHCVSSCPHGLLGAK

*******:* ***********:**..** *****:********************:****

ENSP00000267101_Hsap/1-1342 GPIYKYPDVQNECRPCHENCTQGCKGPELQDCLGQTLVLIGKTHLTMALTVIAGLVVIFM

ENSSHAP00000006754_Shar/1-1328 GLIYKYPDAHRECLPCHENCTQGCNGPELHDCLGQPQAISSKTHVAVGLAVVAGLIVITL

* ******.:.** **********:****:*****. .: .***:::.*:*:***:** :

ENSP00000267101_Hsap/1-1342 MLGGTFLYWRGRRIQNKRAMRRYLERGESIEPLDPSEKANKVLARIFKETELRKLKVLGS

ENSSHAP00000006754_Shar/1-1328 ALLLTLLYLRGRKIQKKRAMRRYLERGESLEPLDPGEKANKVLARIFKETELRKLKVLGS

* *:** ***:**:*************:*****.************************

ENSP00000267101_Hsap/1-1342 GVFGTVHKGVWIPEGESIKIPVCIKVIEDKSGRQSFQAVTDHMLAIGSLDHAHIVRLLGL

ENSSHAP00000006754_Shar/1-1328 GVFGTVHKGIWIPEGESIKIPVCIKVIEDRSGRQSFQAVTDHMLAIGSLDHTHIVRLLGL

*********:*******************:*********************:********

ENSP00000267101_Hsap/1-1342 CPGSSLQLVTQYLPLGSLLDHVRQHRGALGPQLLLNWGVQIAKGMYYLEEHGMVHRNLAA

ENSSHAP00000006754_Shar/1-1328 CPGSSLQLVTQFLPLGSVLDHVRQHRGALGPQLLLNWAVQIAKGMYYLEEHGMVHRNLAA

***********:*****:*******************.**********************

ENSP00000267101_Hsap/1-1342 RNVLLKSPSQVQVADFGVADLLPPDDKQLLYSEAKTPIKWMALESIHFGKYTHQSDVWSY

ENSSHAP00000006754_Shar/1-1328 RNVLLKSPSQVQVADFGVADLLPPDDKQLLHSEAKTPIKWMALESIHFGKYTHQSDVWSY

******************************:*****************************

ENSP00000267101_Hsap/1-1342 GVTVWELMTFGAEPYAGLRLAEVPDLLEKGERLAQPQICTIDVYMVMVKCWMIDENIRPT

ENSSHAP00000006754_Shar/1-1328 GVTIWELMTFGDIPYKGLRLAEVPDLLEKGERLAQPQICTIDVYMVMVKCWMIDENIRPT

***:******* ** ********************************************

ENSP00000267101_Hsap/1-1342 FKELANEFTRMARDPPRYLVIKRESGPGIAPGPEPHGLTNKKLEEVELEPELDLDLDLEA

ENSSHAP00000006754_Shar/1-1328 FKELANEFTRMARDPPRYLVIKRDSGSGLPPGAEIPALTDKELEEV--EPELELDMEL--

***********************:**.*:.**.* .**:*:**** ****:**::*

ENSP00000267101_Hsap/1-1342 EEDNLATTTLGSALSLPVGTLNRPRGSQSLLSPSSGYMPMNQGNLGESCQESAVSGSSER

ENSSHAP00000006754_Shar/1-1328 EEEELA-STLGSALSLPVGTLSRPRGSQSFLSPSSGYMPMNQSNIGGARQGSVTRGSGEQ

**::** :*************.*******:************.*:* : * *.. **.*:

ENSP00000267101_Hsap/1-1342 CPRPVSLHPMPRGCLASESSEGHVTGSEAELQEKVSMCRSRSRSRSPRPRGDSAYHSQRH

ENSSHAP00000006754_Shar/1-1328 CPCPSSLLQNPRGRLTSESSEGRGTSSEAELQEAESLCG----SRSPRPRGDSAYHSQRH

** * ** *** *:******: *.******* *:* *****************

ENSP00000267101_Hsap/1-1342 SLLTPVTPLSPPGLEEEDVNGYVMPDTHLKGTPSSREGTL-SSVGLSSVLGTE-EEDEDE

ENSSHAP00000006754_Shar/1-1328 SLLTPLTPLITPGLEEEDVNGYVMPDTHTKGIPSSRDGTLSSSVGISSILGTEEEEDEDD

*****:*** .***************** ** ****:*** ****:**:**** *****:

ENSP00000267101_Hsap/1-1342 EYEYMNRRRRHSPPHPPRPSSLEELGYEYMDVGSDLSASLGSTQSCPLHPVPIMPTAGTT

ENSSHAP00000006754_Shar/1-1328 EYEYMNRRRKQSLPRPPRPGSLEELGYEYMDVGSDLSASLGSTHSCPLHPVPIMPVPGTT

*********::* *:****.***********************:***********..***

ENSP00000267101_Hsap/1-1342 PDEDYEYMNRQRDGGGPGGDYAAMGACPASEQGYEEMRAFQGPGHQAPHVHYARLKTLRS

ENSSHAP00000006754_Shar/1-1328 PDEDYEYMNRRHGGGTSGSDYAAMGACPAAEQGYEEMGSFQSPGHHAPCIHDAHLKPLQT

**********::.** .*.**********:******* :**.***:** :* *:**.*::

ENSP00000267101_Hsap/1-1342 LEATDSAFDNPDYWHSRLFPKANAQRT

ENSSHAP00000006754_Shar/1-1328 LEATHSAFDNPDYWHSRLFSKASTQGT

****.**************.**.:* *
